# A Study on Prevalence and Determinants of Ototoxicity During Treatment of Childhood Cancer (SOUND): Protocol for a Prospective Study

**DOI:** 10.2196/34297

**Published:** 2022-04-07

**Authors:** Franciscus A Diepstraten, Annelot JM Meijer, Martine van Grotel, Sabine LA Plasschaert, Alexander E Hoetink, Marta Fiocco, Geert O Janssens, Robert J Stokroos, Marry M van den Heuvel-Eibrink

**Affiliations:** 1 Princess Máxima Center for Pediatric Oncology Utrecht the Netherlands; 2 Department of Otorhinolaryngology and Head & Neck Surgery University Medical Center Utrecht Utrecht University Utrecht the Netherlands; 3 University Medical Center Utrecht Brain Center Utrecht University Utrecht the Netherlands; 4 Mathematical Institute Leiden University Leiden the Netherlands; 5 Department of Biomedical Data Science Section Medical Statistics Leiden University Medical Center Leiden the Netherlands; 6 Department of Radiation Oncology University Medical Center Utrecht Utrecht the Netherlands

**Keywords:** pediatrics, ototoxicity, audiometry, antibiotics, diuretics, radiotherapy, solid tumors, neuro-oncology, audiology, cancer, children

## Abstract

**Background:**

Some children with central nervous system (CNS) and solid tumors are at risk to develop ototoxicity during treatment. Up to now, several risk factors have been identified that may contribute to ototoxicity, such as platinum derivates, cranial irradiation, and brain surgery. Comedication, like antibiotics and diuretics, is known to enhance ototoxicity, but their independent influence has not been investigated in childhood cancer patients. Recommendations for hearing loss screening are missing or vary highly across treatment protocols. Additionally, adherence to existing screening guidelines is not always optimal. Currently, knowledge is lacking on the prevalence of ototoxicity.

**Objective:**

The aim of the Study on Prevalence and Determinants of Ototoxicity During Treatment of Childhood Cancer (SOUND) is to determine the feasibility of audiological testing and to determine the prevalence and determinants of ototoxicity during treatment for childhood cancer in a national cohort of patients with solid and CNS tumors.

**Methods:**

The SOUND study is a prospective cohort study in the national childhood cancer center in the Netherlands. The study aims to include all children aged 0 to 19 years with a newly diagnosed CNS or solid tumor. Part of these patients will get audiological examination as part of their standard of care (stratum 1). Patients in which audiological examination is not the standard of care will be invited for inclusion in stratum 2. Age-dependent audiological assessments will be pursued before the start of treatment and within 3 months after the end of treatment. Apart from hearing loss, we will investigate the feasibility to screen patients for tinnitus and vertigo prevalence after cancer treatment. This study will also determine the independent contribution of antibiotics and diuretics on ototoxicity.

**Results:**

This study was approved by the Medical Research Ethics Committee Utrecht (Identifier 20-417/M). Currently, we are in the process of recruitment for this study.

**Conclusions:**

The SOUND study will raise awareness about the presence of ototoxicity during the treatment of children with CNS or solid tumors. It will give insight into the prevalence and independent clinical and cotreatment-related determinants of ototoxicity. This is important for the identification of future high-risk patients. Thereby, the study will provide a basis for the selection of patients who will benefit from innovative otoprotective intervention trials during childhood cancer treatment that are currently being prepared.

**Trial Registration:**

Netherlands Trial Register NL8881; https://www.trialregister.nl/trial/8881

**International Registered Report Identifier (IRRID):**

DERR1-10.2196/34297

## Introduction

Each year around 600 children are newly diagnosed with cancer in the Netherlands [1]. Over the past decades, cancer diagnostics and treatment have improved, resulting in an overall survival up to about 80% in high-income countries [2]. Due to the increased survival, more awareness has been raised for sequelae of childhood cancer treatment. A serious direct and late effect of treatment includes ototoxicity, which involves the destruction of cochlear structures leading to hearing loss, tinnitus (ear ringing), or vertigo (dizziness) [3,4]. Ototoxicity in childhood may seriously affect speech development and social and neurocognitive skills, subsequently leading to a reduced quality of life [5-7].

It is known that certain types of cancer treatment including platinum agents, cranial irradiation, and brain surgery can induce or enhance ototoxicity [3,8]. Platinum agents have been used successfully for treatment of solid and central nervous system (CNS) tumors [8]. Platinum accumulates in the inner ear and resides here for months to years after treatment [9]. It forms crosslinks with DNA, leading to a transcription and replication blockage and extensive production of reactive oxygen species (ROS). ROS cause inflammation and apoptosis, especially in the outer hair cells, stria vascularis, and spiral ganglion cells [10,11]. Up to 70% of platinum-treated children develop irreversible hearing loss and eventually 40% of them even need hearing aids at an early stage [12-14]. During cancer treatment aminoglycosides, glycopeptides, and diuretics are often prescribed as supportive care therapy [15-18], but knowledge is lacking on their contribution to hearing loss development in pediatric patients. Until now, the relation between supportive care treatment and ototoxicity has only been studied in small pediatric cancer patient cohorts with a retrospective design.

Children diagnosed with CNS tumors or head and neck tumors are often treated with high irradiation doses or surgery [19]. Irradiation of normal ear structures cannot always be avoided, leading to middle and inner ear damage or vascular insufficiency of the ear [20-22]. Mechanical damage of ear structures caused by surgical resection of tumors may also lead to hearing loss [23]. It may also be possible that the tumor itself causes hearing loss. Currently, standardized approaches for audiological monitoring during and shortly after therapy are lacking in clinical practice. Beside this, adherence to scheduling of audiological examinations is sometimes flawed, as the focus of clinicians is on cancer diagnostics and rapid start of treatment, rather than on monitoring side effects. Sometimes patients are too ill to undergo audiological testing. Furthermore, age-appropriate audiological tests are often not applied, and standardized tinnitus and vertigo screening is often not implemented. Standardized audiological monitoring is important as it will aid in the timely detection of symptomatic or asymptomatic hearing loss and early referral to an audiologist. In certain circumstances, alternative cancer treatment options may be considered depending on the child’s diagnosis and evidence-based alternative treatments.

In the Princess Máxima Center for Pediatric Oncology, the Study on Prevalence and Determinants of Ototoxicity During Treatment of Childhood Cancer (SOUND) was started in January 2021. We intend to invite 600 patients in this study over a period of 24 months. This number is based on the number of children diagnosed with solid and CNS tumors at our institute. The results of the SOUND study will provide information on the prevalence of hearing loss, tinnitus, and vertigo in a prospective cohort of children with solid and CNS tumors. It will also give insight into the determinants of ototoxicity, especially the contribution of aminoglycosides, glycopeptides, and diuretics. Furthermore, the results will indicate whether it is at all feasible to perform standardized audiological examinations in every childhood cancer patient with a potential risk of ototoxicity. Eventually, this study may serve as a solid basis for the selection of patients at risk that will benefit from innovative otoprotective interventions in the future.

## Methods

### Study Objectives

The primary objective of the study is to investigate the prevalence of hearing loss after cancer treatment in a prospective cohort of pediatric CNS and solid tumor patients. Secondary objectives focus on examining the prevalence of tinnitus and vertigo after cancer treatment, identifying clinical determinants for ototoxicity, and investigating the feasibility of testing patients at risk for ototoxicity with standardized audiological examinations.

### Study Design and Setting

We will perform a national prospective cohort study in the Netherlands. This study is embedded in the Princess Máxima Center, a national health care center that has been set up to treat all children diagnosed with cancer. Collaboration with the audiological department of Wilhelmina Children’s Hospital in the Netherlands has been established to perform audiological testing of children of all ages. The selection, invitation, and inclusion of patients takes place in the Princess Máxima Center. The audiological assessment is age-adjusted, according to recently published guidelines [24]. It will be performed before the start of treatment, during cancer treatment according to treatment protocols, and within 3 months after the end of treatment (Table 1). Information about the screening outcome of the neonatal hearing screening is retrieved for all patients [25].

**Table 1:** Stratification and time point for audiological examination based on treatment.

	Audiological assessment			
Treatment	Stratum	Before start treatment	During treatment^a^	Within 3 months after treatment
Platinum agents (cisplatin, carboplatin, oxaliplatin)	1	✓^b^	✓	✓
CNS^c^/ENT^d^ irradiation	1^e^	✓		✓
CNS/ENT surgery	1^e^	✓		✓
No platinum agents, CNS/ENT irradiation, or CNS/ENT treatment	2	✓		✓

^a^Time points for audiological assessments during treatment are based on recommendations in the childhood cancer treatment protocol.

^b^The checkmark indicates that the assessment was performed at this time point.

^c^CNS: central nervous system.

^d^ENT: ear-nose-throat.

^e^Patients are included in stratum 1 if audiological examinations are part of standard care; otherwise, they are included in stratum 2.

### Study Population

Children aged 0 to 19 years who will be diagnosed with a CNS or solid tumor between January 2021 and January 2023 are eligible for participation in the study. They will be divided in two strata (Table 1). Stratum 1 consists of patients treated with platinum agents (cisplatin, carboplatin, or oxaliplatin), CNS/ear-nose-throat (ENT) irradiation, or CNS/ENT surgery, and audiological examinations are part of standard care. Stratum 2 consists of patients not treated with therapies described in stratum 1 or any patients in which audiological examinations are not part of standard care. The independent contribution of any comedication on ototoxicity development will be studied in stratum 2.

### Recruitment and Informed Consent

Pediatric oncologists from the Princess Máxima Center will select patients for invitation. For patients in whom audiological examinations are not standard of care, written informed consent will be obtained from parents/guardians (patients) according to good clinical practice regulations. Withdrawal is possible at any time during the study without providing a reason.

### Study Procedures

All patients will be examined by standard audiological examinations (Table 2), which always includes otoscopic inspection of the ears and tympanometry. Depending on the age of the child, (a combination of) brainstem-evoked response audiometry (BERA), distortion product otoacoustic emissions (DPOAEs), visual reinforcement audiometry (VRA), conditioned play audiometry, and extended high-frequency pure tone audiometry (PTA) will be applied. Anamnestic screening for tinnitus and vertigo will be performed during audiological examinations as a standard procedure for patients 8 years and older and 10 years and older, respectively [24,26,27]. We will schedule at least two visits during the study period, including a baseline visit before the start of cancer treatment to exclude pre-existent hearing loss and a follow-up visit within 3 months after the end of cancer treatment.

Otoscopy is used for inspection of the external auditory canal and tympanic membrane, which focuses on accumulation of cerumen, infections, and perforations of the tympanic membrane [28,29]. Tympanometry is routinely applied as an indicator of conductive hearing loss due to middle ear pathology. Static air pressure is varied systematically in the ear canal while an acoustic stimulus of 226 or 1000 Hz is delivered. This procedure measures the ability of the middle ear system to transfer low-frequency acoustic energy to the cochlea as a function of static air canal pressure [30]. Abnormal functioning of the middle ear due to a thickened tympanic membrane; presence of middle ear fluid, for example, caused by acute otitis media; or dysfunction of the Eustachian tube can be detected [31].

BERA will be performed for objectively measuring hearing thresholds to quantify the type and severity of hearing loss [32]. Electric activity of the VIII nerve and its central connections are evoked by a broadband click stimulus or frequency-specific stimulus through a headphone, insert phone, or bone conductor. The responses can be measured by electrodes placed on the scalp and is represented as a registration of measured electrical potentials as a function of time after stimulus presentation, characterized by reproducible peaks at specific time delays (latencies). No active cooperation is required, but a prerequisite is that the patient is asleep or lies in a relaxed position to avoid artifacts due to muscle activity.

Evoked otoacoustic emissions (OAEs) are sounds generated within the inner ear after presentation of a stimulus. OAEs are related to the nonlinear behavior of the cochlea, often referred to as the cochlear amplifier. A clear relationship exists between the presence of normal OAEs and functional outer hair cells, which implies that OAEs can only be evoked in ears with normal hearing thresholds or very mild hearing loss (<25 dB HL) [33,34]. Different types of OAEs exist, but often DPOAEs and transiently evoked OAEs (TEOAEs) are measured. TEOAEs are evoked by using a click stimulus and frequency range up to 4 kHz, in contrast to the DPOAEs that are evoked by using pairs of primary tones with particular intensity. The frequency range that can be monitored is much larger than with TEOAEs and ranges up to 10 kHz [31,33,34]. As ototoxicity will start in the high frequencies, measuring DPOAEs is preferred in addition to TEOAEs in our study.

**Table 2:** Audiological assessments per age category.^a^

Assessment	Age categories			
	0-6 months	6 months-3 years	3-5 years	5-18 years
Check eligibility	✓^b^	✓	✓	✓
Determine stratum	✓	✓	✓	✓
Check treatment plan	✓	✓	✓	✓
Demographic data	✓	✓	✓	✓
Audiological examination				
Anamnesis^c^/case history	✓	✓	✓	✓
Otoscopy	✓	✓	✓	✓
Tympanometry	✓	✓	✓	✓
Otoacoustic emissions	✓	✓		
Brainstem-evoked response audiometry	✓			
Visual reinforcement audiometry		✓		
Conditioned play audiometry			✓	
(Extended high-frequency) pure tone audiometry				✓
Anamnestic tinnitus questions				✓^d^
Anamnestic vertigo questions				✓^e^

^a^The listed audiological examination per age category provides an indication. Due to clinical circumstances or developmental status of the patients, another more appropriate test might be chosen.

^b^The checkmark indicates the assessment was given for these age groups.

^c^During anamnesis children/parents are asked for hearing loss in the past, results of the neonatal hearing screening, ear-nose-throat surgery in the past, and family members with (heredity) hearing loss.

^d^Tinnitus screening will be performed for children 8 years and older.

^e^Vertigo screening will be performed for children 10 years and older.

VRA and conditioned play audiometry are both subjective tests based on a conditioning procedure. For VRA, the child is conditioned to make a head turn and to look at a “reward,” for example, a short movie or picture, every time a frequency-specific stimulus is presented [35,36]. For conditioned play audiometry, children are conditioned to perform a motivation enhancing task when an auditory stimulus is presented, for example, putting a block into a box [31,35]. Frequencies up to 8 kHz can be measured, but for ototoxicity monitoring, the range is preferably extended to the high frequencies up to 12 kHz, as in an early stage, ototoxicity may affect the high frequencies [37].

PTA is a subjective behavioral measurement of hearing thresholds. Conventional PTA measures the faintest tone a person can hear at selected frequencies. Hearing thresholds are obtained via air conduction (0.25-8 kHz) by using headphones and via bone conduction (0.5-4 kHz) by using a vibrating transducer. Additionally, extended high-frequency hearing thresholds may be determined up to 12 to 14 kHz, depending on the age of the child [31,34,38].

Anamnestic screening for tinnitus and vertigo will be performed during audiological assessments as a standardized procedure. Regarding tinnitus, children 8 years and older will be asked whether they hear noise in their ears. If the answer is yes, questions follow on the type of noise (ringing, buzzing, humming, whistling, sea noise, banging, blowing sound, like a machine, clicking, beep, like the wind, or like cars), the moment of onset (dates, after which chemotherapy course), laterality (left ear, right ear, or both), pitch (high vs low), perceived loudness (graded according to a Likert scale 0-5) at the peak moment, duration (intermittent, most, some of the time, or continuous), annoyance degree (always annoyed, seldom annoyed, very annoyed, little annoyed), and severity with regard to causing worry (not at all, slightly, sometimes, more severely) [26]. Regarding vertigo, children 10 years and older will be asked whether they ever get dizzy. If the answer is yes, questions follow on the moment of onset (dates, after which chemotherapy course), presence of light-headedness (yes/no), tendency to fall (yes/no), experiencing spinning or turning objects (yes/no), sensation of turning or spinning (yes/no), loss of balance when walking or running (yes/no), duration (intermittent, most/some of the time, or continuous), and severity (graded according to a Likert scale 0-5) [27].

### Speech Audiometry

In case of hearing loss at the end of cancer treatment, speech audiometry is recommended to test the ability to hear and understand speech. Several lists of words are offered to the child through headphones or a free field loudspeaker. The child is asked to repeat the words. The number of correctly repeated phonemes or words are recorded. The results are registered in a speech audiogram. The test reveals the amount of speech discrimination at various stimulus intensities and provides an indication on the influence of hearing loss on communication. Different test procedures are available, including determination of the ear’s ability to discriminate speech in silence or in different types of noise. Speech audiometry in children is often used as a diagnostic test. In addition, the test can be useful to evaluate the outcome of an intervention with hearing aids [31,34,39].

### Sample Size

A power analysis based on two-sided CIs for one proportion has been performed. The Clopper-Pearson exact formula for computing binomial CIs was used. The method is based on the cumulative probabilities of the binomial distribution rather than an approximation [40,41]. A sample size of 367 patients produces a two-sided 95% CI with a width (distance between lower and the upper limit for the CI) equal to 1% when the sample size proportion is 35%. PASS software (NCSS, LLC) has been used for computing the sample size.

### Outcomes

#### Baseline Characteristics

Baseline variables will be collected including age; gender; weight; height; medical and audiological history; solid/CNS tumor type; presence of neurofibromatosis (yes/no); hydrocephalus (yes/no); which national or international cancer treatment protocol will be applied; and whether a patient receives treatment with CNS/ENT surgery, CNS/ENT irradiation, or platinum agents.

#### Primary Outcome Measures

The primary outcome is the prevalence of hearing loss at the end of cancer treatment in a prospective cohort of children with CNS and solid tumors. Hearing loss is classified according to the Muenster classification [42]. The definition of sensorineural hearing loss at the end of treatment will be >40 dB at 4 kHz (corresponding to Muenster grade 2b) [42] measured by BERA, VRA, conditioned play audiometry, or PTA. The SIOP Boston criteria will be applied as a second grading for a reliable determination of hearing loss [43].

#### Secondary Outcome Measures

Secondary outcomes include the prevalence of tinnitus and vertigo, and the treatment components that may be associated to hearing loss, tinnitus, and/or vertigo development. Tinnitus and vertigo will be measured by the aforementioned study procedures. The definition of tinnitus at the end of treatment will be “a sensation of a noise in the ear or head when no apparent source for the noise is evident” [26]. The definition of vertigo is “an abnormal sensation of motion,” which can occur in the absence of motion or when a motion is sensed inaccurately [27]. Treatment components of included patients that will be collected include total cumulative dose of cisplatin, carboplatin, and oxaliplatin; irradiation type (photon or proton) and total dose in Gray (especially on inner ear structures); type of CNS/ENT surgery, cerebral shunt, or Ommaya reservoir; aminoglycoside type, levels, and total cumulative dose; glycopeptide type, levels, and total cumulative dose; and diuretic type and cumulative dose. Liver and kidney function will be measured during treatment. These results will be collected and considered, as platinum agents, antibiotics, and diuretics are (partially) excreted by liver and kidneys.

Secondary outcomes also include the feasibility of standard care audiological testing of all patients with solid and CNS tumors treated with platinum, CNS/ENT irradiation, or CNS/ENT surgery before and after childhood cancer therapy by using standardized diagnostic tests, timing, and frequency of audiological evaluations in this population (stratum 1).

### Statistical Analysis

Continuous variables will be reported with medians and ranges while categorical variables with percentages. To assess differences between patients with and without hearing loss, tinnitus, or vertigo, chi-square and Student *t* tests will be used for categorical and continuous variables, respectively. In case of violation of normality, Mann-Whitney *U* tests will be used.

A logistic regression model will be estimated to investigate the effect of risk factors such as platinum use and cumulative dose, type of CNS/ENT surgery, type of CNS/ENT irradiation and dose, type of comedication and its dose and levels, age at diagnosis, and gender on ototoxicity at the end of treatment. To quantify the effect of prognostic factors on the risk of ototoxicity at the end of treatment, odds ratios along with 95% CIs will be estimated. Statistical analysis will be performed with SPSS, version 26.0.0.1. (IBM Corp) [44].

In the presence of missing data, imputing techniques are used [45].

### Ethics Approval

The study protocol has been approved by the Clinical Research Committee of the Princess Máxima Center and by the Medical Research Ethics Committee Utrecht (Identifier 20-417/M), and has been registered in the Netherlands Trial Register (NL8881).

## Results

Inclusion started on January 4, 2021. Participant recruitment and data collection are still ongoing. Patient inclusion will be finished on January 1, 2023.

## Discussion

### Study Rationale

Ototoxicity is a serious adverse event of childhood cancer treatment. However, the prevalence of hearing loss, tinnitus, and vertigo during and shortly after treatment has never been studied in large prospective pediatric oncology patient cohorts. To date, the association of clinical characteristics and treatment components on the development of ototoxicity is not fully understood. Therefore, we will investigate the feasibility of testing all patients with a potential risk on ototoxicity with standardized audiological examinations. The prevalence and determinants of hearing loss, tinnitus, and vertigo will be investigated in a prospective cohort of childhood cancer patients. Accurate audiological testing and timely detection of ototoxicity will identify novel compounds at risk for ototoxicity and create awareness, which may lead to improved patient care and improved quality of life. Furthermore, we aim to collect high-quality data on the relation between disease status, type of treatment, and audiological functioning in children with solid and CNS tumors before and shortly after treatment. In the future, these data could be used to identify children who are at risk and might benefit from otoprotective agents that are currently under development.

### Limitations

First, it is important to realize that pediatric oncology patients can be critically ill at the time of presentation at the hospital. In that case, it may not be feasible to perform baseline audiological examination. For these selected children, we decided to use audiological anamnesis to exclude any type of pre-existent hearing loss. Second, it is possible that patients will receive medication (eg, antibiotics and diuretics) in shared care centers, which are not reported and may lead to underreporting comedication.

## Abbreviations

BERA: brainstem-evoked response audiometry
CNS: central nervous system
DPOAE: distortion product otoacoustic emission
ENT: ear-nose-throat
OAE: otoacoustic emission
PTA: pure tone audiometry
ROS: reactive oxygen species
SOUND: Study on Prevalence and Determinants of Ototoxicity During Treatment of Childhood Cancer
TEOAE: transiently evoked otoacoustic emission
VRA: visual reinforcement audiometry

## Data Availability

Data sharing is not applicable, as no data set is currently available for analysis in this study protocol. A data transfer agreement between the Wilhelmina Children’s Hospital/University Medical Center Utrecht and the Princess Máxima Center is available to transfer audiological data.
